# Reductive Dealkylation of Anisole and Phenetole: Towards Practical Lignin Conversion

**DOI:** 10.1002/ejoc.201101015

**Published:** 2011-08-11

**Authors:** Zea Strassberger, Stefania Tanase, Gadi Rothenberg

**Affiliations:** [a] Van't Hoff Institute for Molecular Sciences, University of AmsterdamScience Park 904, 1098 XH Amsterdam, The Netherlands, Fax: +31-20-525-5604 E-mail: g.rothenberg@uva.nl

**Keywords:** Heterogeneous catalysis, Sustainable chemistry, Reduction, Biomass, Bulk chemicals

## Abstract

We present and develop alternative catalysts for biomass conversion and specifically lignin conversion into aromatics. Unlike the conventional CoMo and NiMo formulations, our catalysts can convert low-sulfur feedstocks. A set of five magnesia–alumina mixed oxides were screened in the hydrodealkylation of alkyl phenyl ethers as lignin model compounds. The typical selectivity to phenol is 30–75 %. Interestingly, we saw that the more basic the catalyst, the higher the selectivity for phenol. The results concur with the formation of phenoxide (PhO^–^) and RH_3_^+^ fragments on the catalyst surface. These can then react with H^+^ and H^–^ species formed by the hydrogen dissociation on the MgO surface, giving phenol and hydrocarbons. We conclude that magnesia–alumina mixed oxides are attractive candidates for catalyzing lignin breakdown. These catalysts are highly stable, inexpensive, and readily available.

## Introduction

Lignin, the glue that holds trees together, is the most abundant natural resource of aromatic compounds.^1^–^3^ In that respect, it is a far more advanced resource than crude oil. This is because lignin already contains aromatic functional groups. Crude oil must first undergo cracking and then reformation, both of which are energy intensive and costly.^4^–^9^ Thus, catalytic conversion of lignin into high-value aromatics is not only politically attractive but also an economically viable option.^6^,^10^,^11^

The problem is that lignin is typically over-functionalized. Its polymeric structure must first be broken down to dimeric and monomeric components.^12^,^13^ These must then be transformed into the desired functional aromatics. Interestingly, changing the hydrocarbon feedstock from petroleum and coal into biomass also requires new types of catalysts. CoMo and NiMo are typically used for catalyzing crude oil hydrodesulfurization (HDS),^14^,^15^ but these refinery catalysts rely on feedstocks with high sulfur content. The lower sulfur content of biomass causes catalyst deactivation via reduction of sulfided Co or Ni followed by coking.^16^ This can be prevented by adding sulfur donor compounds to the feed,^17^,^18^ which are then converted into H_2_S, but it is a “degenerate” solution. Thus, new catalysts are needed for these new feedstocks.^10^

With this in mind, we searched for an alternative hydrodeoxygenation (HDO) catalyst that needs no sulfiding and is capable of converting low-sulfur feedstocks. Here we report a new type of mixed magnesia–alumina catalyst for the reductive dealkylation of anisole and phenetole, two lignin model compounds, and discuss the pros and cons of their application.

## Results and Discussions

As lignin model compounds, we chose two simple alkyl phenyl ethers, namely, anisole (methoxybenzene) and phenetole (ethoxybenzene). These are also two important and actual breakdown products of the lignin structure. As catalysts, we used five different mixed alumina–magnesia oxides (catalysts **A**–**E**, see Table 1). In a typical reaction [Equation (1)], a solution of the alkyl phenyl ether was treated with 40 bar H_2_ at 350 °C for 3 h in the presence of the catalyst (10 wt.-%). The main reactions observed were hydrodealkylation and alkyl rearrangement [Equations (2), (3), and (4)]. The latter is very interesting, as it leads to the formation of new C–C bonds. Until now, only acidic heterogeneous catalysts have been reported, including cation-exchanged montmorillonites,^19^ Nafion,^20^ and zeolites. To investigate the effect of basic sites, we used MgO^21^ and MgO–Al_2_O_3_ mixed oxides at various Mg/Al ratios. In the latter case, the presence of Al^3+^ ions is expected to change the acidic character of MgO.


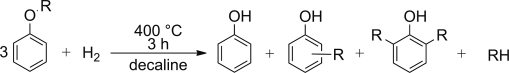
(1)


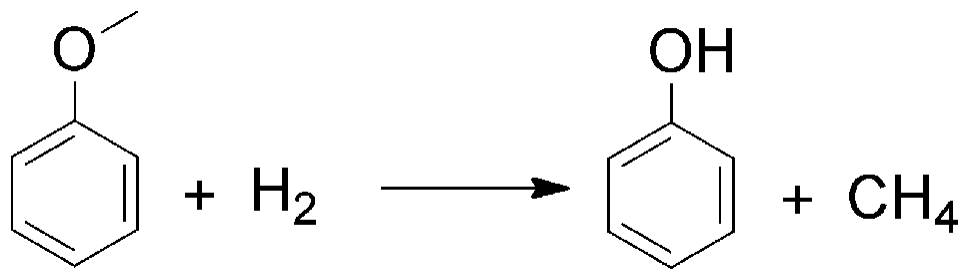
(2)


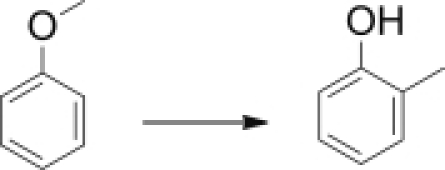
(3)


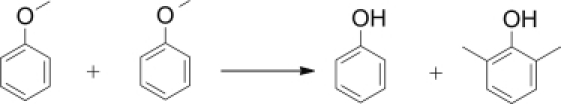
(4)

**Table 1:** Catalysts tested in hydrodealkylation

Entry	Catalyst	% MgO	% Al_2_O_3_
1	**A**	0	100
2	**B**	60	40
3	**C**	66	34
4	**D**	75	25
5	**E**	80	20

Both anisole and phenetole gave high conversions with 100 % alumina. For anisole, the main products of the demethylation were phenol, *ortho*-cresol, and 2,6-xylenol (Table 2). *ortho*-Cresol is obtained by the isomerization of anisole [Equation (2)]. We did not observe any *meta*-cresol. This is expected if we consider that the *meta* position is thermodynamically the most favored for the substitution in phenol rings, whereas the *ortho* position is kinetically preferred due to its reactivity towards electrophilic substitution. The formation of 2,6-xylenol can be explained by the disproportionation reaction between two *ortho*-cresol molecules [Equation (4)].^22^

**Table 2:** Product distribution for anisole and phenetole conversion using catalysts **A**–**E**^[a]^

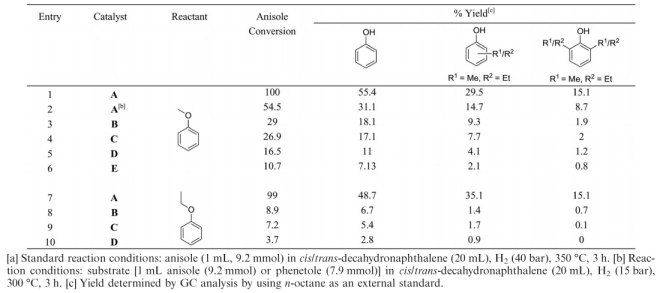

**Scheme 1:**
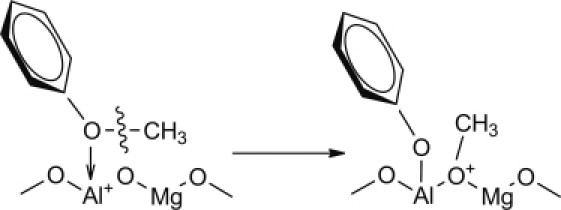
Proposed interaction of phenol with the Al_2_O_3_–MgO support.

The high selectivity towards phenol can be interpreted by considering the adsorption of anisole, a weak Lewis base, onto the acidic Al_2_O_3_ sites. This makes the anisole prone to nucleophilic attack. The most reactive nucleophilic site is the oxygen bound to magnesium, which can attack the methyl group of the anisole molecule. This gives phenoxide (PhO^–^) and CH_3_^+^ fragments on the surface (Scheme 1). These fragments can then react with H^+^ and H^–^ species formed by hydrogen dissociation on the MgO surface, giving phenol and methane [Equation (2)]. Participation of H^+^ and H^–^ is documented in based-catalyzed hydrogenation.^23^ Our studies show, however, that anisole can also be converted into phenol with good selectivity in the absence of hydrogen. Therefore, we do not rule out the possibility of a nucleophilic interaction between CH_3_^+^ cations and an O^2–^ anion from the MgO surface.^23^ This would give a formate surface species, which may decompose into CO and H_2_ at high temperatures.^24^

Recent studies have shown that Lewis acid sites play a key role in the formation of CH_3_^+^ fragments.^25^ Indeed, we also see that Al_2_O_3_ is necessary for activating anisole. Table 2 shows that lowering the Al_2_O_3_ content decreases anisole conversion. MgO alone does not catalyze the conversion of anisole. We also studied the rearrangement of phenetole. As seen in Table 2, phenetole is less reactive than anisole. Nevertheless, a similar trend is observed for both substrate conversion and product distribution.

Table 2 also shows a definite synergistic behavior increasing the selectivity in the conversion of anisole. The more basic the catalyst, the higher the selectivity for phenol. The price is a sharp lowering of the conversion of anisole. Such a decrease can be explained by considering that anisole molecules bind to the Lewis acid sites. When the number of these sites (Al_2_O_3_ sites) decreases, they are rapidly saturated and subsequent anisole molecules can interact only via hydrogen bonding.^25^ Therefore, less anisole molecules will be activated, lowering the conversion.

Studies on the effect of temperature and hydrogen pressure were carried out by using catalyst **A** (Table 2, Entry 2). The conversion of anisole decreased at lower temperature and lower hydrogen pressure. However, the selectivity towards phenol remained unchanged, although less methylated and dimethylated products were observed.

**Figure 1:**
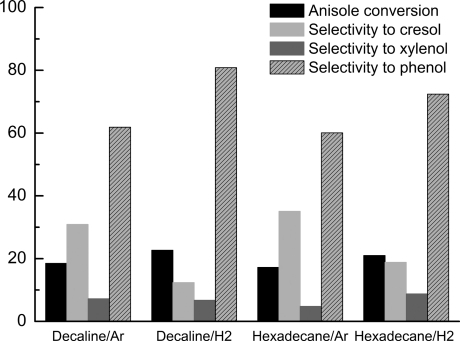
Effect of the solvent on the conversion of anisole.

Hydrogen affects the distribution of products when using both decaline and hexadecane as solvents (Figure 1). The yields of *ortho*-cresol and xylenol are higher compared with those obtained under an atmosphere of argon. However, the selectivity towards phenol is lower in hexadecane compared with decaline. This suggests the involvement of decaline as a hydrogen donor. The use of decaline as a solvent gives a similar conversion of anisole for both argon and hydrogen atmospheres. Higher yields of *ortho*-cresol andxylenol are achieved.

In another set of experiments, we studied the role of the pretreatment temperature on 2:1 MgO–Al_2_O_3_ (catalyst **C**). We expected that the molecules covering the surface would desorb successively according to their interaction strength with the surface sites. The evolution of water and carbon dioxide continues up to 800 K for MgO.^23^ Consequently, stronger basic sites should form at higher temperatures. Table 3 shows indeed that anisole conversion increases slightly with the pretreatment temperature, but the product distribution remains unchanged.

**Table 3:** Temperature pretreatment effects on conversion and yield[a]

Entry	Activation temp.	Anisole conversion	Phenol	% Yield[b]*o/p*-Cresol	2,6-Xylenol
1	r.t.	23.6	15	6.8	1.9
2	200	26.9	17.1	7.7	2.1
3	400	29.8	19.6	8.1	2
4	600	29.5	19.1	8.1	2.3

[a] Standard reaction conditions: anisole (1 mL, 9.2 mmol) in *cis*/*trans*-decahydronaphthalene (20 mL), H_2_ (40 bar), 350 °C, 3 h.

[b] Yield determined by GC analysis by using *n*-octane as an external standard.

## Conclusions

Magnesia–alumina mixed oxides are attractive candidates for catalyzing lignin breakdown reactions. These catalysts are highly stable, inexpensive, and readily available. Pure alumina is not the preferred catalyst because it shows low selectivity for phenol, but 60:40 magnesia–alumina shows high selectivity for phenol at reasonable conversion. These mixed oxides can be used alternatives for petrochemical feedstock catalysts in the conversion of biomass and bio-oils.

## Experimental Section

**Materials and Instrumentation:** Gas chromatography (GC) analysis was performed by using an Interscience GC-8000 gas chromatograph equipped with a flame ionization detector (FID), 14 % cyanopropylphenyl and 86 % dimethyl polysiloxane capillary column (Rtx-1701, 30 m; 25 mm ID; 1 μm df). Samples for GC analysis were diluted in pentane (1 mL). Reactants and products were quantified by using octane as an external standard. GC conditions: isotherm at 50 °C (2 min); ramp at 2 °C min^–1^ to 70 °C; ramp at 70 °C min^–1^ to 140 °C; ramp at 10 °C min^–1^ to 260 °C; isotherm at 260 °C (2 min). All reactions were performed under 40 bar of hydrogen using a 40-mL stainless steel autoclave. Unless otherwise noted, all chemicals used were purchased from commercial sources and used as received. All products were identified by comparing their GC retention times to those of authentic samples. The Al_2_O_3_–MgO mixed oxides were provided by Eurosupport.^26^

**Procedure for Alkyl Transfer of Anisole and Ethyl Benzene Ether:** Screening of the different supports (0.1 g) was performed in a 40-mL stainless steel autoclave. A solution of anisole (1 mL, 9.2 mmol) in *cis*/*trans*-decahydronaphthalene (20 mL) was charged into the reactor. The pressure was increased to 40 bar with H_2_, after which the reactor was heated at the desired reaction temperature (300–350 °C). All the supports were tested at 350 °C for 3 h. The effect of the reaction temperature and pressure (300 °C and 15 bar H_2_) was studied only on catalyst **A**. After the reaction, the reactor was cooled down to room temperature by using an ice bath. Liquid samples were analyzed by GC.

**Procedure for Catalyst Activation:** Each catalyst sample was heated at 200 °C under an atmosphere of N_2_ flow for 2 h prior catalytic tests. This precaution was taken to avoid any differences on the catalyst surface related to water or other species deposition that could interfere. Different temperatures of activation were also tested, from r.t. to 600 °C always under N_2_ flow. Table 3 shows only a slight increase in the conversion of anisole and the yield of phenol. Because the difference were minor and because the procedure time consuming, we used 200 °C as a standard temperature of activation.

## References

[1] Calvo-Flores FG, Dobado JA (2010). ChemSusChem.

[2] Sannigrahi P, Pu YQ, Ragauskas A (2010). Curr. Opin. Environ. Sustainability.

[3] van Haveren J, Scott EL, Sanders J (2008). Biofuels Bioprod. Biorefining.

[4] Albertazzi S, Basile F, Brandin J, Einvall J, Fornasari G, Hulteberg C, Sanati A, Trifiro F, Vaccari A (2009). Energy Fuels.

[5] Calderone VR, Shiju NR, Ferre DC, Rothenberg G (2011). Green Chem..

[6] Keim W (2010). Pet. Chem..

[7] Shah A, Fishwick R, Wood J, Leeke G, Rigby S, Greaves M (2010). Energy Environ. Sci..

[8] Song CS (2003). Catal. Today.

[9] Zagaglia P (2010). Energy Econ..

[10] Zakzeski J, Bruijnincx PCA, Jongerius AL, Weckhuysen BM (2010). Chem. Rev..

[11] Zakzeski J, Weckhuysen BM (2011). ChemSusChem.

[12] Hatakeyama H, Hatakeyama T (2010). Biopolymers: Lignin.

[13] Vanholme R, Morreel K, Ralph J, Boerjan W (2008). Curr. Opin. Plant Biol..

[14] Dhar GM, Srinivas BN, Rana MS, Kumar M, Maity SK (2003). Catal. Today.

[15] Leyva C, Rana MS, Trejo F, Ancheyta J (2007). Ind. Eng. Chem. Res..

[16] Laurent E, Centeno A, Delmon B, Delmon B, Froment GF (1994). Catalyst Deactivation 1994.

[17] Echard M, Leglise J (2001). Catal. Lett..

[18] Leliveld RG, van Dillen AJ, Geus JW, Koningsberger DC (1998). J. Catal..

[19] Tateiwa J, Nishimura T, Horiuchi H, Uemura S (1994). J. Chem. Soc. Perkin Trans. 1.

[20] Kaspi J, Olah GA (1978). J. Org. Chem..

[21] Montero JM, Gai P, Wilson K, Lee AF (2009). Green Chem..

[22] Grabowska H, Zawadzki M, Syper L (2004). Appl. Catal. A.

[23] Hattori H (1995). Chem. Rev..

[24] Lazo ND, Murray DK, Kieke ML, Haw JF (1992). J. Am. Chem. Soc..

[25] Popov A, Kondratieva E, Goupil JM, Mariey L, Bazin P, Gilson JP, Travert A, Mauge F (2010). J. Phys. Chem. C.

[26] http://www.eurosupport.nl/.

